# Supplementary material for: Drosophila nicotinic acetylcholine receptor subunits and their native interactions with insecticidal peptide toxins
Source: eLife. 2022 May 16;11:e74322. doi: 10.7554/eLife.74322 (PMC9110030; doi:10.7554/eLife.74322)
Supplement: Supplementary file 1. [file elife-74322-supp1.docx]

| **10-day old flies** | | | | | | | | | |
| --- | --- | --- | --- | --- | --- | --- | --- | --- | --- |
| **number** | **receptor** | **series 1** | | **series 2** | | **series 3** | | **average** | **standard deviation** |
|  | **subunit** | **no of flies with phenotype** | **percentage** | **percentage** | **actual change** | **percentage** | **actual change** | **percent** | **STEV** |
| 1 | *nAChRα1* | 12 | 24 | 14 | 28 | 14 | 28 | 26.666667 | 2.309401077 |
| 2 | *nAChRα2* | 10 | 20 | 9 | 18 | 11 | 22 | 20 | 2 |
| 3 | *nAChRα3* | 0 | 0 | 1 | 2 | 0 | 0 | 0.6666667 | 1.154700538 |
| 4 | *nAChRα4* | 0 | 0 | 0 | 0 | 0 | 0 | 0 | 0 |
| 5 | *nAChRα5* | 8 | 16 | 7 | 14 | 7 | 14 | 14.666667 | 1.154700538 |
| 6 | *nAChRα6* | 0 | 0 | 0 | 0 | 2 | 4 | 1.3333333 | 2.309401077 |
| 7 | *nAChRα7* | 1 | 2 | 1 | 2 | 0 | 0 | 1.3333333 | 1.154700538 |
| 9 | *nAChRβ2* | 0 | 0 | 2 | 4 | 0 | 0 | 1.3333333 | 2.309401077 |
| 10 | *nAChRβ3* | 6 | 12 | 9 | 18 | 7 | 14 | 14.666667 | 3.055050463 |
| 11 | *W^1118^* | 0 | 0 | 0 | 0 | 0 | 0 | 0 | 0 |

## Supplementary Figure 1. Abdomen phenotype.
