# Supplementary material for: Drosophila nicotinic acetylcholine receptor subunits and their native interactions with insecticidal peptide toxins
Source: eLife. 2022 May 16;11:e74322. doi: 10.7554/eLife.74322 (PMC9110030; doi:10.7554/eLife.74322)
Supplement: Supplementary file 2. [file elife-74322-supp2.docx]

| **10-day old flies** | | | | | | | | | |
| --- | --- | --- | --- | --- | --- | --- | --- | --- | --- |
| **number** | **receptor** | **series 1** | | **series 2** | | **series 3** | | **average** | **standard deviation** |
|  | **subunit** | **percentage** | **actual change** | **percentage** | **actual change** | **percentage** | **actual change** | **percent** | **STEV** |
| 1 | *nAChR α1* | 30 | 37.5 | 40 | 50 | 50 | 71.4 | 53 | 17.2 |
| 2 | *nAChR α2* | 40 | 50 | 40 | 50 | 50 | 71.4 | 57.1 | 12.4 |
| 3 | *nAChR α3* | 0 | 0 | 20 | 25 | 30 | 42.9 | 22.6 | 21.5 |
| 4 | *nAChR α4* | 70 | 87.5 | 80 | 100 | 60 | 85.7 | 91.1 | 7.8 |
| 5 | *nAChR α5* | 60 | 75 | 70 | 87.5 | 50 | 71.4 | 78 | 8.4 |
| 6 | *nAChR α6* | 40 | 50 | 50 | 62.5 | 50 | 71.4 | 61.3 | 10.8 |
| 7 | *nAChR α7* | 70 | 87.5 | 80 | 100 | 70 | 100 | 95.8 | 7.2 |
| 9 | *nAChR β2* | 80 | 100 | 80 | 100 | 60 | 85.7 | 95.2 | 8.2 |
| 10 | *nAChR β3* | 80 | 100 | 80 | 100 | 70 | 100 | 100 | 0 |
| 11 | *W^1118^* | 80 | 100 | 80 | 100 | 70 | 100 | 100 | 0 |

## Supplementary Figure 2. Climbing ability.
