# Supplementary material for: Drosophila nicotinic acetylcholine receptor subunits and their native interactions with insecticidal peptide toxins
Source: eLife. 2022 May 16;11:e74322. doi: 10.7554/eLife.74322 (PMC9110030; doi:10.7554/eLife.74322)
Supplement: Supplementary file 3. [file elife-74322-supp3.docx]

|  |  |  | **Survival (% of pupae formed after injection)** | | | | |
| --- | --- | --- | --- | --- | --- | --- | --- |
|  | **Strain** | **Injected cpd** | **Rep1** | **Rep2** | **Rep3** | **Average** | **Standard Deviation** |
| CONTROL | *w^1118^* | 2.5 nmol/g Hv1a | 0 | 0 | 0 | 0 | 0 |
| CONTROL - *Cas9* lines | *THattP40* | 2.5 nmol/g Hv1a | 0 | 0 | 0 | 0 | 0 |
| CONTROL - *Cas9* lines | *THattP2* | 2.5 nmol/g Hv1a | 0 | 0 | 0 | 0 | 0 |
| nAChR CRISPR mutant | *nAChRα1* | 2.5 nmol/g Hv1a | 25 | 0 | 0 | 8.33 | 14.43 |
| nAChR CRISPR mutant | *nAChRα2* | 2.5 nmol/g Hv1a | 25 | 0 | 0 | 8.33 | 14.43 |
| nAChR CRISPR mutant | *nAChRα3* | 2.5 nmol/g Hv1a | 0 | 0 | 0 | 0 | 0 |
| nAChR CRISPR mutant | *nAChRα4* | 2.5 nmol/g Hv1a | 25 | 33.33 | 66.67 | 41.67 | 22.05 |
| nAChR CRISPR mutant | *nAChRα5* | 2.5 nmol/g Hv1a | 0 | 0 | 0 | 0 | 0 |
| nAChR CRISPR mutant | *nAChRα6* | 2.5 nmol/g Hv1a | 0 | 0 | 0 | 0 | 0 |
| nAChR CRISPR mutant | *nAChRα7* | 2.5 nmol/g Hv1a | 0 | 0 | 0 | 0 | 0 |
| nAChR CRISPR mutant | *nAChRβ2* | 2.5 nmol/g Hv1a | 25 | 33.33 | 66.67 | 41.67 | 22.05 |
| nAChR CRISPR mutant | *nAChRβ3* | 2.5 nmol/g Hv1a | 0 | 33.33 | 0 | 11.11 | 19.25 |
| Injection CONTROL | *w^1118^* | PBS | 100 | 100 | 100 | 100 | 0 |
|  |  |  |  |  |  |  |  |
| CONTROL | *w^1118^* | 1.25 nmol/g α-Btx | 0 | 0 | 0 | 0 | 0 |
| CONTROL - *Cas9* lines | *THattP40* | 1.25 nmol/g α-Btx | 0 | 0 | 0 | 0 | 0 |
| CONTROL - *Cas9* lines | *THattP2* | 1.25 nmol/g α-Btx | 0 | 0 | 0 | 0 | 0 |
| nAChR CRISPR mutant | *nAChRα1* | 1.25 nmol/g α-Btx | 0 | 0 | 33.33 | 11.11 | 19.25 |
| nAChR CRISPR mutant | *nAChRα2* | 1.25 nmol/g α-Btx | 25 | 0 | 33.33 | 19.44 | 17.35 |
| nAChR CRISPR mutant | *nAChRα3* | 1.25 nmol/g α-Btx | 0 | 0 | 33.33 | 11.11 | 19.25 |
| nAChR CRISPR mutant | *nAChRα4* | 1.25 nmol/g α-Btx | 0 | 33.33 | 0 | 11.11 | 19.25 |
| nAChR CRISPR mutant | *nAChRα5* | 1.25 nmol/g α-Btx | 50 | 66.67 | 66.67 | 61.11 | 9.62 |
| nAChR CRISPR mutant | *nAChRα6* | 1.25 nmol/g α-Btx | 25 | 66.67 | 66.67 | 52.78 | 24.06 |
| nAChR CRISPR mutant | *nAChRα7* | 1.25 nmol/g α-Btx | 50 | 100 | 66.67 | 72.22 | 25.46 |
| nAChR CRISPR mutant | *nAChRβ2* | 1.25 nmol/g α-Btx | 0 | 33.33 | 0 | 11.11 | 19.25 |
| nAChR CRISPR mutant | *nAChRβ3* | 1.25 nmol/g α-Btx | 0 | 0 | 0 | 0 | 0 |
| Injection CONTROL | *w^1118^* | PBS | 100 | 100 | 100 | 100 | 0 |

## Supplementary Figure 3. *Drosophila* larval injection of ω-Hexatoxin-Hv1a & α-Bungarotoxin.
