# Supplementary material for: Drosophila nicotinic acetylcholine receptor subunits and their native interactions with insecticidal peptide toxins
Source: eLife. 2022 May 16;11:e74322. doi: 10.7554/eLife.74322 (PMC9110030; doi:10.7554/eLife.74322)
Supplement: Supplementary file 4. — Peptides from Dα3, Dα5, Dα6, Dα7 and Dβ3 nAChR subunits are listed and found [N] times within individual replicates. Protein domains are marked with: Ed extracellular-, Id Intracellular-, LBD ligand-binding-, and Non-domain localization. The mass-to-charge ratio (m/z) of the precursor ions, the protonated monoisotopic masses, the theoretical MH+ masses in Dalton [Da] and peptide modifications are listed. Peptide modifications are listed with: (C) Carbamidomethylation; (N,Q) Deamidation; (H) N-acetylhexosamine (HexNAc); (M) Oxidation. [file elife-74322-supp4.docx]

| **Subunit** | **Accession** | **Sequence** | **Found [N]** | **Domain** | **m/z [Da]** | **MH+ [Da]** | **Theo. MH+ [Da]** | **Modification** |
| --- | --- | --- | --- | --- | --- | --- | --- | --- |
| *nAChRα3* | Q9W3G6 | ATLnYTGR | 2 | LBD | 549.77423 | 1098.54119 | 1098.54258 | H4 or position 2 , 6 |
| *nAChRα5* | Q7KT97 | TVYGQGDDGSIGPIGSTR | 7 | Id | 890.42413 | 1779.84099 | 1779.85078 |  |
| *nAChRα5* | Q7KT97 | TVYGqGDDGSIGPIGSTR | 2 | Id | 890.93036 | 1780.85344 | 1780.83480 | Q5 |
| *nAChRα5* | Q7KT97 | FITDQLR | 7 | Id | 446.74768 | 892.48808 | 892.48869 |  |
| *nAChRα5* | Q7KT97 | KHQILSDVELKER | 1 | Id | 399.47717 | 1594.8869 | 1594.8911 |  |
| *nAChRα5* | Q7KT97 | SSTEYELGLILK | 1 | Id | 676.86945 | 1352.7316 | 1352.7308 |  |
| *nAChR  * | Q7KTF97, Q9VWI9 | LEWNDMNLR | 1 | LBD | 595.78156 | 1190.55583 | 1190.5623 |  |
| *nAChR  * | Q7KTF97, Q9VWI9 | NnGScLYVPPGIFK | 2 | LBD | 884.93384 | 1768.86040 | 1768.85745 | H2, C5 |
| *nAChR  * | Q7KTF97, Q9VWI9 | NnGScLYVPPGIFK | 2 | LBD | 783.88092 | 1566.75456 | 1566.76209 | N2, C5 |
| *nAChR  * | Q7KTF97, Q9VWI9 | nnGScLYVPPGIFK | 2 | LBD | 784.39069 | 1567.77410 | 1567.74611 | N1, N2, C5 |
| *nAChRα6* | Q7KTF9 | ELQFITAR | 4 | Id | 489.27380 | 977.54033 | 977.54146 |  |
| *nAChRα6* | Q7KTF9 | ELqFITAR | 1 | Id | 489.7753 | 978.54332 | 978.52547 | Q3 |
| *nAChRα6* | Q7KTF9 | TADIHEMPPWIK | 1 | Non | 719.36407 | 1437.72087 | 1437.71950 |  |
| *nAChRα6* | Q7KTF9 | TILLSNR | 4 | Id | 408.75095 | 816.49462 | 816.49378 |  |
| *nAChRα6* | Q7KTF9 | ADDEAELIGDWK | 4 | Id | 681.3179 | 1361.61785 | 1361.62195 |  |
| *nAChRα6* | Q7KTF9 | KADDEAELIGDWK | 1 | Id | 745.36469 | 1489.72209 | 1489.71691 |  |
| *nAChRα6* | Q7KTF9 | KTILLSNR | 2 | Id | 472.79971 | 944.59215 | 944.58874 |  |
| *nAChR  α7* | Q7KT97, Q7KTF9, Q9VWI9 | IDITWFPFDDQR | 3 | LBD | 776.87292 | 1552.7386 | 1552.7431 |  |
| *nAChR  α7* | Q7KT97, Q7KTF9, Q9VWI9 | SLLANVLDIDDDFR | 3 | Id | 803.40924 | 1605.8112 | 1605.8119 |  |
| *nAChR α7* | Q7KTF9, Q9VWI9 | IDITWFPFDDqR | 1 | LBD | 777.37311 | 1553.73894 | 1553.7209 | Q11 |
| *nAChRα7* | Q9VWI9 | SLLANVLDIDDDFRcNHR | 1 | Id | 544.01544 | 2173.0399 | 2173.0455 | C15 |
| *nAChRα7* | Q9VWI9 | KQQIQNVELKER | 6 | Id | 504.95410 | 1512.84774 | 1512.84927 |  |
| *nAChRα7* | Q9VWI9 | KQQIQNVELK | 1 | Id | 614.35730 | 1227.70732 | 1227.70556 |  |
| *nAChRα7* | Q9VWI9 | KQqIQNVELKER | 1 | Id | 502.29062 | 1513.8573 | 1513.8333 | N3 |
| *nAChRα7* | Q9VWI9 | QGDDGSVGPVGPAGPVVDGR | 12 | Id | 918.44794 | 1835.88860 | 18335.88823 |  |
| *nAChRα7* | Q9VWI9 | qGDDGSVGPVGPAGPVVDGR | 1 | Id | 918.94897 | 1936.89067 | 1836.87225 | Q1 |
| *nAChRα7* | Q9VWI9 | QQIQNVELK | 1 | Id | 550.30487 | 1099.60246 | 1099.61060 |  |
| *nAChRα7* | Q9VWI9 | EDETSDITR | 1 | Non | 533.23535 | 1065.46343 | 1065.46947 |  |
| *nAChRα7* | Q9VWI9 | mQRPGQVGYEcPPPPSSSSSSASGEK | 1 | Id | 908.41028 | 2723.2163 | 2723.2036 | M1; C11 |
| *nAChRα7* | Q9VWI9 | cASATLPHQPTYYR | 2 | Id | 555.59827 | 1664.7803 | 1664.785 | C1 |
| *nAChRα7* | Q9VWI9 | WITEQLKKEDETSDITR | 2 | Id | 698.01874 | 2092.0417 | 2092.0557 |  |
| *nAChRα7* | Q9VWI9 | WITEQLK | 1 | Non | 459.25516 | 917.50304 | 917.50909 |  |
| *nAChRα7* | Q9VWI9 | cNHRCASATLPHQPTYYR | 1 | Id | 832.89795 | 1664.7886 | 1664.785 | C1 |
| *nAChRβ3* | Q9VPQ8 | VVLPEnGTAR | 1 | LBD | 629.83380 | 1258.66033 | 1258.66376 | H6 or position 8 |

## Supplementary Figure 4. Identified nAChR peptides in pull-downs with α-Bungarotoxin.

Peptides from Dα3, Dα5, Dα6, Dα7 and D nAChR subunits are listed and found [N] times within individual replicates. Protein domains are marked with: Ed extracellular-, Id Intracellular-, LBD ligand*-*binding-, and Non-domain localization. The mass-to-charge ratio (m/z) of the precursor ions, the protonated monoisotopic masses, the theoretical MH^+^ masses in Dalton [Da] and peptide modifications are listed. Peptide modifications are listed with: (C) Carbamidomethylation; (N,Q) Deamidation; (H) N-acetylhexosamine (HexNAc); (M) Oxidation.
