# Supplementary material for: Drosophila nicotinic acetylcholine receptor subunits and their native interactions with insecticidal peptide toxins
Source: eLife. 2022 May 16;11:e74322. doi: 10.7554/eLife.74322 (PMC9110030; doi:10.7554/eLife.74322)
Supplement: Supplementary file 5. — Identified peptides of nAChR subunits which are found in control pull-down samples without α-Bungarotoxin (α-Btx). [file elife-74322-supp5.docx]

## Supplementary Figure 5. Identified nAChR peptides in pull-downs without α-Bungarotoxin.

| **Subunit** | **Accession** | **Sequence** | **Found [N]** | **Domains** | **m/z [Da]** | **MH+ [Da]** | **Theo. MH+ [Da]** | **Modification** |
| --- | --- | --- | --- | --- | --- | --- | --- | --- |
| *nAChRα1* | A0A0B4KGU3 | LFIQILPK | 1 | Ed | 486.31339 | 971.61949 | 917.62882 |  |
| *nAChRα1, α2* | A0A0B4KGU3, P17644 | LYDDLLSNYNR | 1 | Ed | 693.3407 | 1385.674 | 1385.66957 |  |
| *nAChRα2* | P17644 | AIDVQLSDVAK | 2 | Non | 579.823 | 1158.639 | 1158.636 |  |
| *nAChRα2* | P17644 | VVWTPPAIFK | 1 | Ed | 579.3391 | 1157.671 | 1157.671 |  |
| *nAChRα2, β2* | P17644, P25162 | LSQLIEVNLK | 1 | Ed | 386.5676 | 1157.692 | 1157.677 |  |
| *nAChRα4* | A8JNX5 | LVSSGYNNSLPK | 10 | Non | 639.8441 | 1278.661 | 1278.66884 |  |
| *nAChRα4* | A8JNX5 | LVSSGYnNSLPK | 1 | Non | 640.3381 | 1279.669 | 1279.653 | N7 |
| *nAChRα4* | A8JNX5 | LSQLIDVNLK | 2 | LBD | 571.8425 | 1142.678 | 1142.678 |  |
| *nAChRα4* | A8JNX5 | SPILNNPAFSHSK | 1 | Non | 471.24591 | 1411.72318 | 1411.73284 |  |
| *nAChRα4* | A8JNX5 | RPTYNFETSK | 3 | Non | 621.80939 | 1242.61150 | 1242.61133 |  |
| *nAChRα4* | A8JNX5 | RPTYnFETSK | 3 | Non | 622.3102 | 1243.613 | 1243.595 | N5 |
| *nAChRα4* | A8JNX5 | LYDDLLSNYNK | 2 | LBD | 679.3725 | 1357.64910 | 1357.66342 |  |
| *nAChRα4* | A8JNX5 | RPTYNFETSKLLLK | 1 | Non | 621.8079 | 1242.609 | 1242.611 |  |
| *nAChRβ1* | P04755 | NKNFVDLSDYWK | 2 | Ed | 643.8068 | 1286.600 | 1286.605 |  |
| *nAChRβ1* | P04755 | NFVDLSDYWK | 4 | Ed | 643.8044 | 1286.602 | 1286.605 |  |
| *nAChRβ1* | P04755 | nFVDLSDYWK | 4 | Ed | 644.3093 | 1287.611 | 1287.589 | N1 |
| *nAChRβ1* | P04755 | ILPPTSLVLPLIAK | 1 | Non | 737.9825 | 1474.96074 | 1474.96071 |  |
| *nAChRβ1* | P04755 | ATEAVEFIAEHLR | 5 | Id | 495.92743 | 1485.76773 | 1485.76962 |  |
| *nAChRβ1* | P04755 | VWKPDIVLFNNADGNYEVR | 1 | Ed | 750.3779 | 2249.119 | 2249.135 |  |
| *nAChRβ2* | P25162 | LYDDLLSNYNR | 3 | Ed | 693.34070 | 1385.67412 | 1385.66957 |  |
| *nAChRβ2* | P25162 | LSqLIEVNLKNqVMTTNLWVK | 1 | Ed | 825.1133 | 2473.325 | 2473.3370 | Q3; Q12 |
| *nAChRβ2* | P25162 | LSqLIEVNLK | 2 | Ed | 386.5676 | 1157.688 | 1157.678 | Q3 |

Identified peptides of nAChR subunits which are found in control pull-down samples without α-Bungarotoxin (α-Btx).
