# Supplementary material for: Drosophila nicotinic acetylcholine receptor subunits and their native interactions with insecticidal peptide toxins
Source: eLife. 2022 May 16;11:e74322. doi: 10.7554/eLife.74322 (PMC9110030; doi:10.7554/eLife.74322)
Supplement: Supplementary file 6. [file elife-74322-supp6.docx]

| **Knockouts** | | | | |
| --- | --- | --- | --- | --- |
| **nAChR subunit** | **gRNA target name** | **gRNA sequence (NGG)** | **oligonucleotides name (Forward/Reverse)** | **oligonucleotides sequence** |
| *nAChRα1* | Da1_111(+) | 5’CGGAGATGTAGTAGTCCTGCAGG3’ | 41_Da1_111_F | 5’GTCGCGGAGATGTAGTAGTCCTGC3’ |
|  |  |  | 42_Da1_111_R | 5’AAACGCAGGACTACTACATCTCCG3’ |
|  | Da1_126(-) | 5’CCTGCAGGTCGATGCCCACCTCG3’ | 43_Da1_126_F | 5’GTCGCGAGGTGGGCATCGACCTGC3’ |
|  |  |  | 44_Da1_126_R | 5’AAACGCAGGTCGATGCCCACCTCG3’ |
| *nAChRα2* | Da2_99 (+) | 5’GCTCCTCTGCGAAACCGTTCAGG3’ | 45_Da2_99_F | 5’GTCGCTCCTCTGCGAAACCGTTC3’ |
|  |  |  | 46_Da2_99_R | 5’AAACGAACGGTTTCGCAGAGGAG3’ |
| *nAChRα3* | Da3_18(+) | 5’GTCCGGACGCCAGATGTGATCGG3’ | 49_Da3_18_F | 5’GTCGTCCGGACGCCAGATGTGAT3’ |
|  |  |  | 50_Da3_18_R | 5’AAACATCACATCTGGCGTCCGGA3’ |
| *nAChRα4* | Da4_19(+) | 5’TTGTTGCGACGAACCATACTTGG3’ | 53_Da4_19_F | 5’GTCGTTGTTGCGACGAACCATACT3’ |
|  |  |  | 54_Da4_19_R | 5’AAACAGTATGGTTCGTCGCAACAA3’ |
| *nAChRα5* | Da5_232(-) | 5’CCGGGGATCTTCAAGTCGACGTG3’ | 57_Da5_232_F | 5’GTCGCACGTCGACTTGAAGATCCC3’ |
|  |  |  | 58_Da5_232_R | 5’AAACGGGATCTTCAAGTCGACGTG3’ |
|  | Da5_251(+) | 5’CGTGCAAGATCGACATCACGTGG3’ | 59_Da5_251_F | 5’GTCGCGTGCAAGATCGACATCACG3’ |
|  |  |  | 60_Da5_251_R | 5’AAACCGTGATGTCGATCTTGCACG3’ |
| *nAChRα6* | Da6_70(+) | 5’CGTATTCTTCTTTCCCGGCATGG3’ | 61_Da6_70_F | 5’GTCGCGTATTCTTCTTTCCCGGCA3’ |
|  |  |  | 62_Da6_70_R | 5’AAACTGCCGGGAAAGAAGAATACG3’ |
| *nAChRα7* | Da7_1226(+) | 5’CATTGACCACCGGACGCTCCAGG3’ | 63_Da7_1226_F | 5’GTCGCATTGACCACCGGACGCTCC3’ |
|  |  |  | 64_Da7_1226_R | 5’AAACGGAGCGTCCGGTGGTCAATG3’ |
| *nAChRβ1* | Db1_2(+) | 5’TGGAGTCTTCCTGCAAATCCTGG3’ | 67_Db1_2_F | 5’GTCGTGGAGTCTTCCTGCAAATCC3’ |
|  |  |  | 68_Db1_2_R | 5’AAACGGATTTGCAGGAAGACTCCA3’ |
| *nAChRβ2* | Db2_955(+) | 5’TCAGACCTAACCAAACCGTCAGG3’ | 71_Db2_955_F | 5’GTCGTCAGACCTAACCAAACCGTC3’ |
|  |  |  | 72_Db2_955_R | 5’AAACGACGGTTTGGTTAGGTCTGA3’ |
| *nAChRβ3* | Db3_466(+) | 5’CTTTGAAGTCCAGCGAGGTCTGG3’ | 75_Db3_466_F | 5’GTCGCTTTGAAGTCCAGCGAGGTC3’ |
|  |  |  | 76_Db3_466_R | 5’AAACGACCTCGCTGGACTTCAAAG3’ |
| **C-terminal tagging** | | | | |
| **nAChR subunit** | **gRNA target name** | **gRNA sequence (NGG)** | **oligonucleotides name (Forward/Reverse)** | **oligonucleotides sequence** |
| *nAChRα6* | Da6_181(+) | 5’TTGCACGATTATGTGCGGAGCGG3’ | 131_Da6_181_F | 5’GTCGTTGCACGATTATGTGCGGAG3’ |
|  |  |  | 132_Da6_181_R | 5’AAACCTCCGCACATAATCGTGCAA3’ |
|  | Da6_176(+) | 5’CCTTATTGCACGATTATGTGCGG3’ | 133_Da6_176_F | 5’GTCGCCTTATTGCACGATTATGTG3’ |
|  |  |  | 134_Da6_176_R | 5’AAACCACATAATCGTGCAATAAGG3’ |

## Supplementary Figure 6. List of gRNAs and oligonucleotides used for cloning.
