# Supplementary material for: Drosophila nicotinic acetylcholine receptor subunits and their native interactions with insecticidal peptide toxins
Source: eLife. 2022 May 16;11:e74322. doi: 10.7554/eLife.74322 (PMC9110030; doi:10.7554/eLife.74322)
Supplement: Supplementary file 7. [file elife-74322-supp7.docx]

| **knockouts** | | | |
| --- | --- | --- | --- |
| **nAChR subunit** | **homology arm** | **oligonucleotides name (Forward/Reverse)** | **oligonucleotides sequence** |
| *nAChRα1* | Da1_LHA | Da1_LHA_F1 | 5’TGGGGCGACAAAATAGCATG3’ |
|  |  | Da1_LHA_R1 | 5’GGGGAAATGGGCCAACAAAT3’ |
| *nAChRα1* | Da1_RHA | Da1_RHA_F1 | 5’GCAGATACTTTCCCAGCAGC3’ |
|  |  | Da1_RHA_R1 | 5’CCGCGTCCTTGACTACTTTG3’ |
| *nAChRα2* | Da2_LHA | Da2_LHA_F1 | 5’ACGAAATGCAAAACCGAGCT3’ |
|  |  | Da2_LHA_R2 | 5’CCCAATTTGACCAACACCGT3’ |
| *nAChRα2* | Da2_RHA | Da2_RHA_F1 | 5’GCGGGCAGAAAGGTAAACAA3’ |
|  |  | Da2_RHA_R1 | 5’TCACCTGATCACCGTCGTAG3’ |
| *nAChRα3* | Da3_LHA | Da3_LHA_F1 | 5’CTCCAGCCGTTCCCAAATCT3’ |
|  |  | Da3_LHA_R1 | 5’CAATCTGTGGGTGGAGCAGT3’ |
| *nAChRα3* | Da3_RHA | Da3_RHA_F1 | 5’CTGCTCGTCGAAGGGAAAGT3’ |
|  |  | Da3_RHA_R1 | 5’GATCCGAGCCAGACTAAGCC3’ |
| *nAChRα4* | Da4_LHA | Da4_LHA_F1 | 5’GATGAACAACAGGGCAGCAA3’ |
|  |  | Da4_LHA_R1 | 5’CAAAACAACAACCGTCACGC3’ |
| *nAChRα4* | Da4_RHA | Da4_RHA_F1 | 5’TTAGAGCGTAACAGTGGGCG3’ |
|  |  | Da4_RHA_R1 | 5’ACGCCTACAAACCGGACAAA3’ |
| *nAChRα5* | Da5_LHA | Da5_LHA_F1 | 5’ACCGCATTCCTGTCGCATAT3’ |
|  |  | Da5_LHA_R1 | 5’CAGGACGACGTTGGCTTACT3’ |
| *nAChRα5* | Da5_RHA | Da5_RHA_F1 | 5’GGATCTTCAAGTCGACGTGC3’ |
|  |  | Da5_RHA_R1 | 5’GAGGGTGTGGCTGGATTTTC3’ |
| *nAChRα6* | Da6_LHA | Da6_LHA_F1 | 5’GTGTACGGGTGTGAGACAGA3’ |
|  |  | Da6_LHA_R1 | 5’TCACACATTGCTTGCCGAAA3’ |
| *nAChRα6* | Da6_RHA | Da6_RHA_F1 | 5’GTCAGTTTCTCGCCCGAATC3’ |
|  |  | Da6_RHA_R1 | 5’CCGAGAGTTGACTGTAGCCA3’ |
| *nAChRα7* | Da7_LHA | Da7_LHA_F1 | 5’TGTAAACCCTAGCAGTGCCA3’ |
|  |  | Da7_LHA_R1 | 5’TATGATACCGGGTGAGTGCC3’ |
| *nAChRα7* | Da7_RHA | Da7_RHA_F1 | 5’CATCCGGTTTCCATAGGCGA3’ |
|  |  | Da7_RHA_R1 | 5’ACGGAAATCACAATGCCCCT3’ |
| *nAChRβ1* | Db1_LHA | Db1_LHA_F1 | 5’TCATCAACAGCAGGCAGAGA3’ |
|  |  | Db1_LHA_R1 | 5’TGGCAATGAGAGCTTGGAGA3’ |
| *nAChRβ1* | Db1_RHA | Db1_RHA_F1 | 5’CTGCAAATCCTGGCTGTTGT3’ |
|  |  | Db1_RHA_R1 | 5’GTGTGTGTGTGTGTGGTCTC3’ |
| *nAChRβ2* | Db2_LHA | D2b_LHA_F1 | 5’TCAACTCAGGACAGCACACA3’ |
|  |  | D2b_LHA_R1 | 5’ACCACCACTTTCCTAGCTCC3’ |
| *nAChRβ2* | Db2_RHA | D2b_RHA_F1 | 5’CCCATCGCAACTTGTAGTCG3’ |
|  |  | D2b_RHA_R1 | 5’CATTCGTCCAGGTAAGTGCG3’ |
| *nAChRβ3* | Db3_LHA | Db3_LHA_F1 | 5’AACGGTTCCGATGACTTCCT3’ |
|  |  | Db3_LHA_R1 | 5’TGAGCATGTTGAGTTCGCAG3’ |
| *nAChRβ3* | Db3_RHA | Db3_RHA_F1 | 5’TCCTTCGTCCTCTCCTTCGT3’ |
|  |  | Db3_RHA_R1 | 5’TTCTGCGGGAAACTACGACC3’ |

## Supplementary Figure 7. List of oligonucleotides used for amplification from genomic DNA.

**Supplementary File 7. Continued, List of oligonucleotides used for amplification from genomic DNA.**

| **C-terminal tagging** | | | |
| --- | --- | --- | --- |
| **nAChR subunit** | **homology arm** | **oligonucleotides name (Forward/Reverse)** | **oligonucleotides sequence** |
| *nAChRα6* | Da6_RHA | 74_Da6_RHA_F_Gen | 5’GGGTTTCTGTTCTTGCGCTG3’ |
|  |  | 75_Da6_RHA_R_Gen | 5’GCCCTGCTGATTTGTTTGCT3’ |
| *nAChRα6* | Da6_LHA | 76_Da6_LHA_F_Gen | 5’CCGATGCTTCCGACGTATCC3’ |
|  |  | 77_Da6_LHA_R_Gen | 5’GCCATACTAGCGCATGACTCT3’ |
