# Supplementary material for: Drosophila nicotinic acetylcholine receptor subunits and their native interactions with insecticidal peptide toxins
Source: eLife. 2022 May 16;11:e74322. doi: 10.7554/eLife.74322 (PMC9110030; doi:10.7554/eLife.74322)
Supplement: Supplementary file 8. [file elife-74322-supp8.docx]

| **C-terminal tagging with FSVS** | | | |
| --- | --- | --- | --- |
| **nAChR subunit** | **fragment** | **site** | **oligonucleotides sequence** |
| *nAChRα6* | 31 | 105__Donor_LHA_Da6 | 5’CGGGCTAATTATGGGGTGTCGCCCTGTGCATGCAGAGAATGAAACC3’ |
|  |  | 106_LHA_Da6_linker_R | 5’CCTTGCACGATTATGTGCGGAGCTGAGAGCAGCACCGTAACCG3’ |
|  | 32 | 107_linkerTag_F | 5’GCTCTCAGCTCCGCACATAATCGTGCAAGGATCCGGCGGAGGGGGC3’ |
|  |  | 108_RHDa6_Tag_R | 5’CTAATTCGAGCGTCCTTACTTTTCGAACTGGGGATGGC3’ |
|  | 33 | 109_TG-RHA_Da6_F | 5’CCCCAGTTCGAAAAGTAAGGACGCTCGAATTAGGCC3’ |
|  |  | 110_Donor_RHDa6 | 5’AAATTTTGTGTCGCCCTTGAACTCGATTTGCGCTGCTTAGCTTCATCTG3’ |
| **C-terminal tagging with FSVS-loxP-3Px3DsRED-loxP using as a template donors with FSVS tags above (fragments 31-33)** | | | |
| **nAChR subunit** | **fragment** | **site** | **oligonucleotides sequence** |
| *nAChRα6* | 34 | 105__Donor_LHA_Da6 | 5’CGGGCTAATTATGGGGTGTCGCCCTGTGCATGCAGAGAATGAAACC3’ |
|  |  | 114_RHDa6_Tag_R | 5’CTTTTCGAACTGGGGATGGCTCCAAGCTCC3’ |
|  | 36 | 155_Marker_F1 | 5’CTTGGAGCCATCCCCAGTTCGAAAAGTAGTAAGGTACCGCGGGTATAAC3’ |
|  |  | 157_Marker_R1 | 5’GGCTCTTCTATATAACTTCGTATAGCATAC3’ |
|  | 36 | 165_Da6_F_marker | 5’ATGCTATACGAAGTTATATAGAAGAGCCTAAGGACGCTCGAATTAGGCC3’ |
|  |  | 110_Donor_RHDa6 | 5’AAATTTTGTGTCGCCCTTGAACTCGATTTGCGCTGCTTAGCTTCATCTG 3’ |

## Supplementary Figure 8. C-terminal tagging of nAChRa6 with FSVS.
