# Supplementary material for: Drosophila nicotinic acetylcholine receptor subunits and their native interactions with insecticidal peptide toxins
Source: eLife. 2022 May 16;11:e74322. doi: 10.7554/eLife.74322 (PMC9110030; doi:10.7554/eLife.74322)
Supplement: Supplementary file 9. [file elife-74322-supp9.docx]

## Supplementary Figure 9. Wet pellet weight of membrane fractions.

|  | **Wet pellet weight** | | | |  |  |
| --- | --- | --- | --- | --- | --- | --- |
| **Spin speed** | **Rep1 (gram)** | **Rep2 (gram)** | **Rep3 (gram)** | **Rep4 (gram)** | **Average (gram)** | **Standard Deviation** |
| 1000 x g | 0.1785 | 0.1767 | 0.1274 | 0.1191 | 0.150425 | 0.027340389 |
| 3000 x g | 0.1202 | 0.0926 | 0.0777 | 0.0817 | 0.09305 | 0.01659646 |
| 5000 x g | 0.0658 | 0.0454 | 0.0506 | 0.0403 | 0.050525 | 0.009541325 |
| 9000 x g | 0.0452 | 0.0388 | 0.0364 | 0.0563 | 0.044175 | 0.007704017 |
| 12000 x g | 0.0217 | 0.0275 | 0.0306 | 0.0222 | 0.0255 | 0.003719543 |
